# Preventing postoperative prosthetic joint dislocation by repairing obturator externus in total hip arthroplasty performed via the posterior approach

**DOI:** 10.1186/s42836-020-00054-4

**Published:** 2020-12-01

**Authors:** Hideki Fujii, Takuya Otani, Yasuhiko Kawaguchi, Tetsuo Hayama, Toshiomi Abe, Motoi Takahashi, Mitsuru Saito

**Affiliations:** Department of Orthopaedic Surgery, The Jikei University School of Medicine, 3-25-8 Nishishinbashi, Minato-ku, Tokyo, 105- 8461 Japan

**Keywords:** Obturator externus, Posterior soft tissues, Postoperative prosthetic joint dislocation, Posterior approach, Total hip arthroplasty

## Abstract

**Background:**

In total hip arthroplasty performed via the posterior approach, repairing the posterior soft tissues is a conventional method for preventing postoperative prosthetic joint dislocation. The aim of this study was to verify whether obturator externus repair played the main role and what was the mechanism of the repair preventing the dislocation.

**Methods:**

Included were 188 patients who underwent primary cementless total hip arthroplasty via the posterior approach. The patients were divided into a repair group (*n* = 94) and a non-repair group (*n* = 94). Patients of repair group received additional obturator externus repair while patients of non-repair group did not. The range of motion of hip joint was assessed before and after operation. Data were compared between the two groups. A *p* value < 0.05 was considered statistically significant.

**Results:**

Before operation and under anesthesia, with regard to internal rotation of hip joint, the mean values of repair and non-repair groups were 24° ± 16/28° ± 15 (*p* = 0.2933). The mean values of the groups were 13° ± 8/15° ± 9 immediately after repair (*p* = 0.5672). Range of internal rotation 1 year after operation were 15° ± 8/19° ± 9 (*p* = 0.0139). Specifically, the values in repair group were lower than those in non-repair group. During a 5-year period of postoperative follow-up, hip joint dislocation occurred in one patient of non-repair group. No dislocation was observed in repair group.

**Conclusion:**

When THA is performed via the posterior approach, repairing the obturator externus may decrease the risk of postoperative prosthetic joint dislocation by reinforcing the posterior soft tissues of the hip joint.

**Level of evidence:**

Therapeutic study, Level IVa.

## Introduction

The posterior approach is the most commonly used approach for total hip arthroplasty (THA), but is associated with a high incidence of postoperative prosthetic joint dislocation (PPJD) [1–3], a serious complication. The early reports showed that repairing the posterior soft tissues could prevent PPJD [4–12], but it is still unclear which posterior structure plays the main role and what is the mechanism by which such repair prevents PPJD.

THA via the posterior approach involves four structures, i.e., (1) the piriformis tendon, (2) the conjoined tendon formed by the superior gemellus, obturator internus, and inferior gemellus, (3) the obturator externus, and (4) the posterior joint capsule. Mihalko et al [13] studied the function of piriformis tendon and conjoined tendon in six human cadavers. Kim et al [14] conducted a retrospective study on 557 patients (670 hips) who underwent primarily THA with preservation of the piriformis, superior gemellus, and obturator internus. Solomon et al [15] described the anatomy of the external rotators in 2010. Gudena et al [16] described the functional anatomy of the obturator externus in 2015. Those studies showed that the piriformis tendon and short external rotators are the important structures for preventing PPJD. Till now, only a few of reports covered the function of obturator externus. To the best of our knowledge, no studies examined the management and repair of the obturator externus. Moreover, the obturator externus tends to retreat into the deeper wound when it is dissected from the greater trochanter, rendering it difficult to identify the stump of obturator externus.

This retrospective study, for the first time, tried to verify whether obturator externus repair played the main role and what was the mechanism in preventing PPJD.

## Patients and methods

The institutional review boards of the participating hospital approved the study. Informed consent was obtained from all patients involved.

We retrospectively set up a repair group and a non-repair group. Repair group comprised 94 patients who were selected from 123 consecutive patients treated between December 2008 and December 2009, coinciding with the period when we repaired the obturator externus. Serving as controls, non-repair group consisted of 94 patients who were selected from 111 consecutive patients treated between November 2007 and November 2008, i.e., the period when we did not repair the obturator externus. Patients in the two groups underwent primary cementless THA via the posterior approach.

The inclusion criteria of our study were primary and secondary hip osteoarthritis, osteonecrosis, dialysis arthropathy and rheumatoid arthritis with a history of pain for 6 months. We excluded patients who had incomplete medical records (*n* = 14 in repair period; *n* = 9 in non-repair period); an early surgery performed on the affected hip (*n* = 4; *n* = 3); a leg 2 cm too short (*n* = 5; *n* = 3) and severe flexion contracture with preoperative flexion ≤30° (*n* = 5; *n* = 2) because incomplete posterior repair. Eventually, 29 and 17 patients were excluded from the aforementioned two study periods, respectively. All operations were either performed directly by the senior author (TO) or under his supervision.

### Surgical technique of repair

**Fig. 1:**
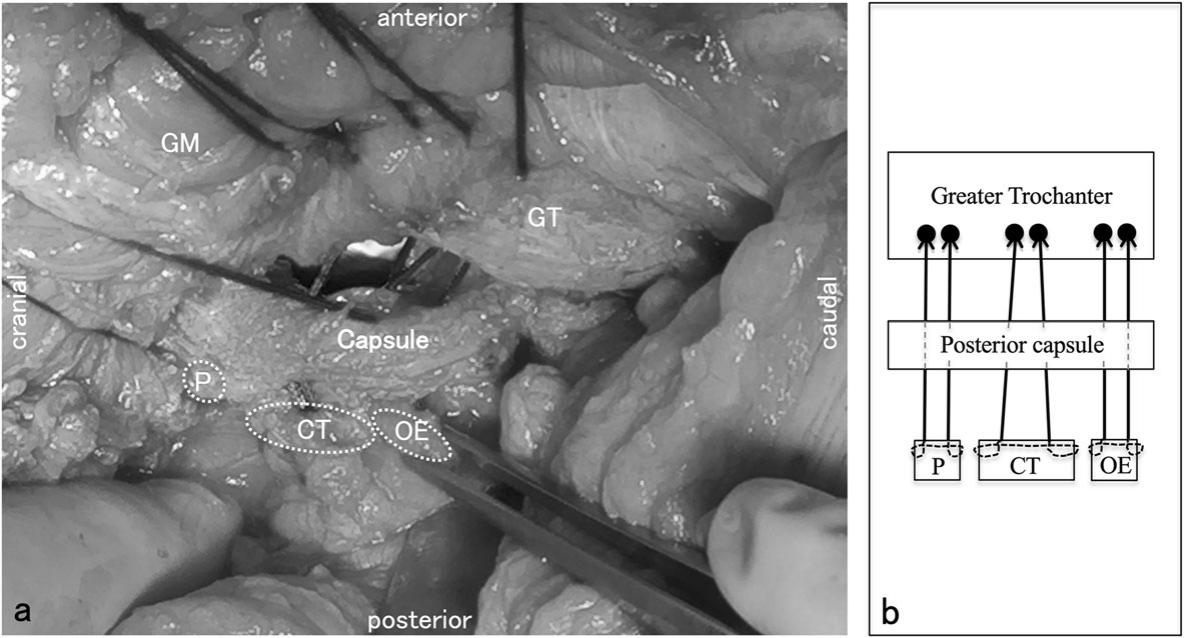
Intraoperatively, the hip joint is placed in the neutral position. GM, gluteus medius; GT, greater trochanter; P, piriformis; CT, conjoined tendon; OE, obturator externus. **a** Showing obturator externus and other posterior soft tissues. **b** The short external rotators and joint capsule were sutured to the posterior insertion of the greater trochanter using three non-absorbable sutures

Operation was performed via the posterior approach. The piriformis, conjoined tendon, and obturator externus were dissected and pulled away from the posterior joint capsule to visualize the greater trochanter. The prosthesis was implanted. The posterior soft tissues were repaired using strong, non-absorbable sutures (Ethibond Excel® size 5; ETHICON; Johnson & Johnson, Somerville, NJ). The tendons were sutured with a locking loop (Kirchmayr-kessler method) [17]. The joint capsule and greater trochanter were reattached with transosseous mattress sutures made behind the greater trochanter and at the anatomical insertion. Three sutures were placed (one suture each for the piriformis, the conjoined tendon, and the obturator externus, respectively.) (Fig. 1a, b). The transected stump of the obturator externus tendon was located distal to the conjoined tendon. Usually, the transection was approximately 10 mm × 5 mm in size, and the obturator externus tendon runs its course along the external surface of the posteroinferior joint capsule toward the deeper wound (Fig. 1a). When suturing, the hip joint was flexed at 30° and placed in the neutral position. The greater trochanter and tendon stump were approximated as much as possible. The optimal windows of abduction and anteversion angles of the acetabular cup were 35–45° and 15–25°, respectively. Bearing combinations included cobalt chrome-on-polyethylene, and femoral heads were either 28 or 32 mm in size. There were no modifications regarding surgical technique other than the presence or absence of obturator externus repair.

### Surgical technique of non-repair group

Patients underwent the same surgical procedures, but one suture was placed for the piriformis and two sutures for the conjoined tendon.

### Postoperative managements

No modifications existed for the postoperative rehabilitation in two groups. The patients were moved in wheelchairs from the first postoperative day, and started ambulatory rehabilitation without weight bearing restrictions from the second postoperative day. Patients were advised to avoid movements or positions increasing the risk of PPJD within the 2 months after surgery. Thereafter, all movements were permitted.

### Outcome evaluation

We retrospectively evaluated the range of motion of hip joint obtained from the patient’s medical records. We assessed the range of internal rotation preoperatively under anesthesia, intraoperatively, immediately after repair, and then 2 months, 4 months, and 1 year after surgery. The range of internal rotation was measured with the hip joint flexed at 60°, and in the neutral position. The angle of flexion was set to 60° during measurement, because our patients’ preoperative range of flexion was ≤90°. We also measured internal rotation under the same conditions. We evaluated hip joint mobility also in terms of preoperative flexion, abduction, and external rotation. We also investigated the incidence of PPJD. All patients were followed up for a minimum of 5 years to identify complications. Functional outcomes were evaluated based on the Japanese Orthopaedic Association scoring system (JOA score), which evaluates function of hip joint on a 100-point scale (40 points for pain, 20 points for ROM, 20 points for ability to walk, and 20 points for activities of daily living). ROM of hip was recorded for flexion, abduction, internal rotation, and external rotation. All assessments were performed by the same senior surgeon who was not involved in the treatment.

### Analysis

Data were expressed as mean ± standard deviation or median and interquartile range. All statistical analyses were performed using the Excel statistical software package (Ekuseru-Toukei 2010; Social Survey Research Information, Tokyo, Japan). Comparisons between repair and non-repair groups were made using Student’s unpaired two-tailed *t*-test. A *p* < 0.05 was considered statistically significant.

## Results

Data on 188 consecutive patients who underwent THA were collected from the Jikei University School of Medicine Hospital. The mean age at the time of surgery was 62 years (range: 21 to 90 years), and the mean body mass index (BMI) was 23 kg/m^2^ (Table 1).

**Table 1:** Demographics

AVE ± SD	Repair group (*n* = 94)	Non-repair group (*n* = 94)	*p*-value
Gender (F:M)	76: 18	77: 17	0.851^a^
Age at surgery (years)	62 ± 10	62 ± 11	0.897^b^
BMI (kg/m^2^)	23.1 ± 3.6	23.1 ± 4.4	0.997^b^
Surgery Side (R:L)	41: 53	54: 40	0.080^a^
Primary Diagnosis			
Primary OA	25	25	
Secondary OA	51	57	0.888^c^
Osteonecrosis	15	10	
Dialysis arthropathy	2	2	
Rheumatoid arthritis	1	0	
Dislocation	0	1	–

n; mean ± sd. ^a^: Fisher’s exact test ^b^: Unpaired Student’s *t*-test ^c^: Chi-square test

Among the 188 patients of both groups, during a 5-year period of postoperative follow-up, hip joint dislocation occurred in one patient of non-repair group. No dislocation was observed in repair group. Posterior dislocation developed 2 months postoperatively when the patient attempted to pick something up. We confirmed failure of posterior repair during repeat surgery on this patient. At the final examination, the mean total JOA score was 80 (35 for pain, 16 for the range of motion, 15 for ambulation, and 14 for ADL).

**Fig. 2:**
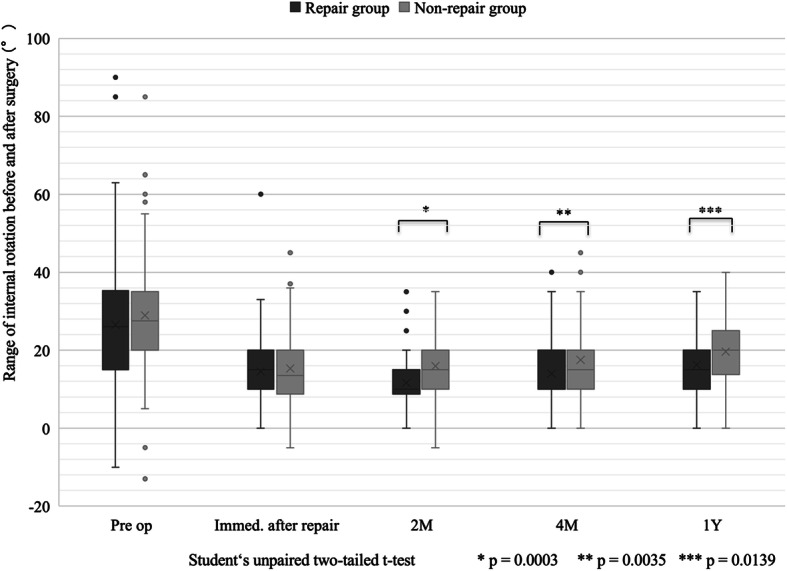
Temporal changes of range of internal rotation before and after surgery in repair and non-repair groups

Before operation and under anesthesia, with regard to internal rotation of hip joint, the mean values of repair and non-repair groups were 24° ± 16 and 28° ± 15 respectively. The mean values of the groups were 13° ± 8 and 15° ± 9 respectively immediately after repair. We found no significant differences between the two groups. Range of internal rotation 2, 4 months, and 1 year after operation were 11° ± 7/16° ± 8, 14° ± 8/17° ± 8, and 15° ± 8/19° ± 9, respectively, with significant differences found between the two groups (*p* = 0.0003; *p* = 0.0035; *p* = 0.0139, respectively). Specifically, the values in repair group were lower than those in non-repair group (Fig. 2).

**Fig. 3:**
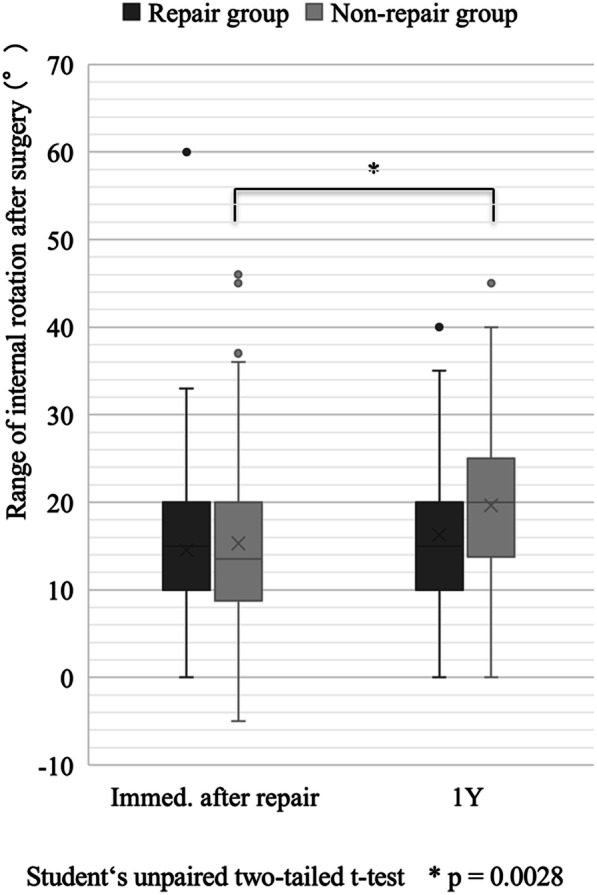
Comparison between repair and non-repair groups with regard to range of internal rotation immediately after surgery and 1 year after surgery

In repair group, there were no significant differences in the range of internal rotation between the time of immediately after surgery (13° ± 8) and 1 year after surgery (15° ± 8) (*p* = 0.172). In non-repair group, we observed a significant difference in the range of internal rotation between the time of immediately after surgery (15° ± 9) and 1 year after surgery (19° ± 9) (*p* = 0.0028) (Fig. 3).

**Fig. 4:**
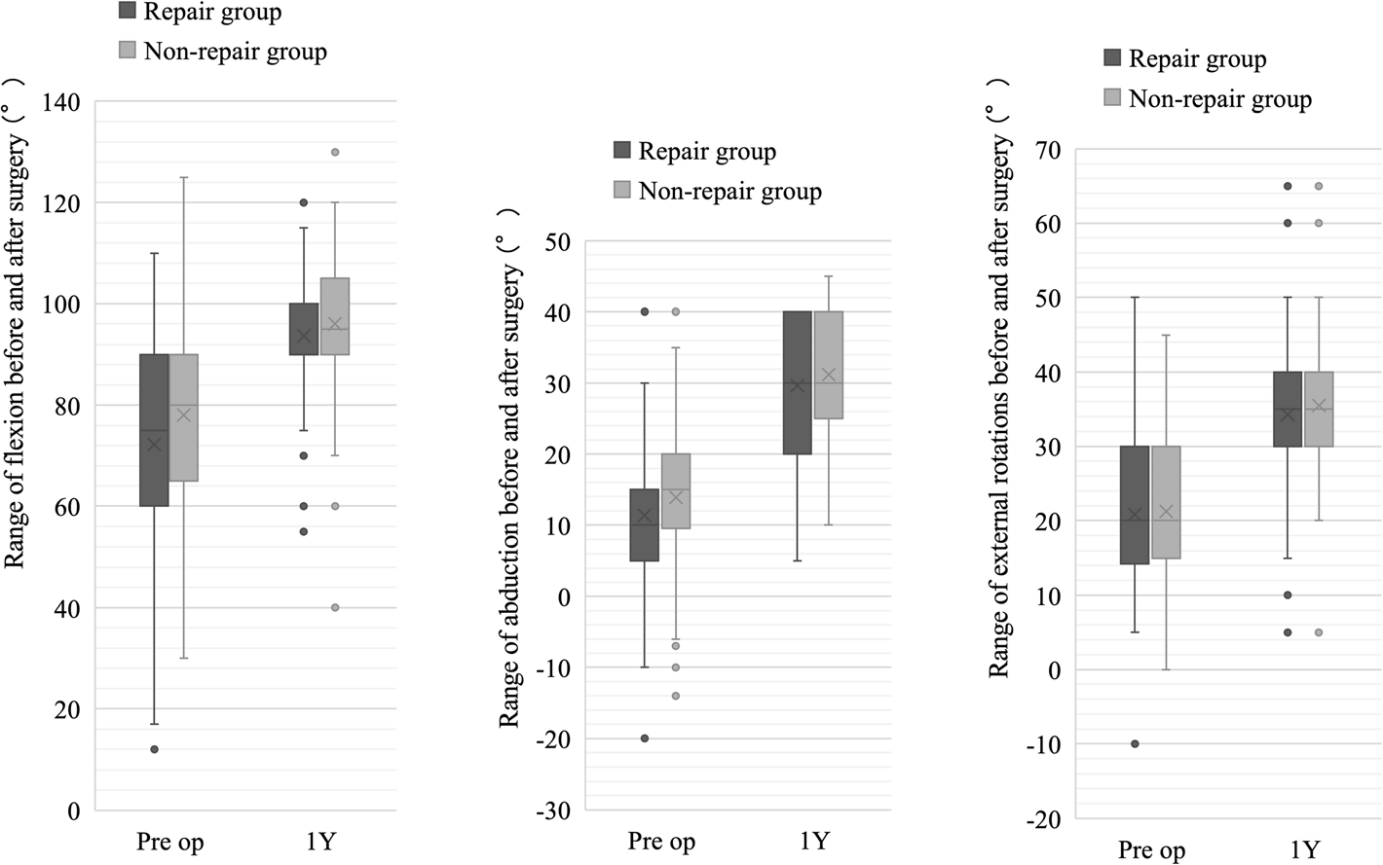
Comparison between the repair and non-repair groups with regard to ranges of flexion, abduction, and external rotation before surgery and 1 year after surgery

The range of motion of hip joint other than internal rotation in the repair and non-repair groups was as follows: preoperatively, flexion 75° ± 20/78° ± 21, abduction 12° ± 8/14° ± 10, external rotation 21° ± 11/21° ± 9; 1 year postoperatively, flexion 94° ± 13/96° ± 16, abduction 28° ± 8/31° ± 8, and external rotation 34° ± 11/35° ± 11. There were no significant differences between the two groups in the range of flexion, abduction, and external rotation preoperatively and 1 year after surgery (Fig. 4).

## Discussion

The posterior approach is reportedly associated with a higher risk of PPJD than other approaches. Moreover, PPJD often occurs when the hip is internally rotated and flexed. Multiple reports suggested that novel surgical approaches, appropriate implant placement, favorable soft tissue balance, and use of large-diameter femoral heads can decrease the risk. Up till now, there have been few reports on the anatomy and function of obturator externus, and its relationship with PPJD.

Our study showed that obturator externus repair, as a part of posterior repair in THA via the posterior approach, did not impair deep flexion of the hip joint. The muscle is capable of reinforcing the restriction of internal rotation when the hip joint is flexed, and this may prevent PPJD.

Kapandji et al [18] demonstrated that the function of the obturator externus was to perform external rotation when the hip joint was flexed. Accordingly, the obturator externus restricts internal rotation when the hip joint is flexed and limits its extension to some extent. Solomon et al [15] anatomically studied the external rotator in 20 cadavers. They described the excursion of the obturator externus based on its course, features, length, and positional changes, but did not mention its function in detail. Gudena et al [16] investigated the functional anatomy of the obturator externus in 18 human cadavers and plastic hip joint models devised by employing Beck et al’s method [19]. They reported that the obturator externus contributed to external rotation and adduction when the hip joint was flexed between 0° and 90°.

Hirano et al [20] focused on the functional anatomy of the obturator externus muscle in an observational study. They examined the relationship to THA performed via the posterior approach. Their results indicated that tension was obvious in the obturator internus when internal rotation was attempted with the hip joint flexed at 0°. However, there was not much tension in this muscle when internal rotation was tried with the hip joint flexed at 90°. In addition, tension on obturator externus increased due to internal rotation when the hip was flexed at either 0° or 90°. These results suggested that the obturator externus plays a much greater role than the obturator internus when restricting internal rotation with the hip joint flexed. Furthermore, Kinoshita et al [21], collaborating with TO and HF, both being the authors of this report, studied six hip joints in three cadavers. They studied the functional anatomy of the obturator externus, including its relationship to the flexion and extension movements and found that the anterior fibers of the obturator externus acted to effect flexion when the hip joint moved from neutral position to 60° of flexion, while the posterior fibers acted to produce extension from 60° to 90° of flexion.

We found that the obturator externus followed a different course compared with other external rotators. We then performed cadaveric studies on the functional anatomy. On the basis of study by Hirano et al [20], we also performed obturator externus repair in addition to repair of the piriformis tendon, conjoined tendon, and joint capsule. Moreover, our cadaveric studies performed in 2014 suggested that the obturator externus could flex and extend the hip joint, and restrict its flexion when the hip was flexed at ≥60°. These findings indicated that increasing obturator externus tension during repair might hinder deep hip joint flexion postoperatively.

We found that when the hip joint was flexed, the range of internal rotation decreased by a mean value of 4° 1 year after surgery due to obturator externus repair. Whether this procedure apparently decreases the onset of PPJD is not certain and further research is warranted. Gudena et al [16] reported that the obturator externus originates from the greater trochanter of the femur, follows a course below the femoral neck, and has a broad insertion into the anterior aspect of the pubic and ischial bones. It acts to stabilize the femoral head in the acetabulum. Thus, additional repair of the obturator externus yields two effects that may contribute to the prevention of PPJD, namely, restricting internal rotation when the hip joint is flexed, and stabilizing the hip joint by increasing concentricity of the femoral head.

In addition to pain relief and gait improvement, functional improvement in mobility is also important for THA. We found a similar effect between repair and non-repair methods in term of ROM, other than postoperative internal rotation. Similar outcomes are also achieved in flexion, abduction and external rotation. Obturator externus repair may not affect other forms of mobility. However, the range of internal rotation is decreased after either repair or non-repair of the muscle. This might be partially ascribed to the fact that the detachment of the external rotator tendons from the greater trochanter and reattachment possibly resulted in the shortening of the tendons. What is more, there may be lengthening of the leg or an increased hip joint offset due to the effects of implant placement. These factors increase tension in the external rotators and may decrease the range of internal rotation. During surgery, it is important to cleanly detach the external rotators from the greater trochanter and avoid shortening of the external rotators. Another topic for future research is determining the position at which the external rotators should be repaired, thereby preventing PPJD and simultaneously ensuring a good range of internal rotation.

Our study has some limitations. This study was of a retrospective design and the surgeries were performed in different periods. It was not a blinded and random study and biases in the results are unavoidable. In addition to suturing the obturator externus, other unidentifiable factors, such as differences between the repair and non-repair methods, could not be ruled out.

## Conclusion

In performing THA via the posterior approach, repairing the obturator externus may decrease the risk of PPJD by reinforcing the posterior soft tissues of the hip joint.

## Data Availability

This study was carried out in the Hospital of the Jikei University School of Medicine (3–25-8 Nishi-Shinbashi Minato-ku, Tokyo, Japan). The datasets used and/or analyzed during the current study are available from the corresponding author on reasonable request.

## Abbreviations

JOA score: Japanese Orthopaedic Association scoring system
ROM: Range of motion
THA: Total hip arthroplasty
